# A secure data sharing scheme based on broadcast signcryption in data center

**DOI:** 10.1371/journal.pone.0335757

**Published:** 2025-11-10

**Authors:** Runyuan Dong, Yang Li, Liangyu Zhu

**Affiliations:** The Second Monitoring and Application Center, CEA, Xi’an, China; Cardiff Metropolitan University - Llandaff Campus: Cardiff Metropolitan University, UNITED KINGDOM OF GREAT BRITAIN AND NORTHERN IRELAND

## Abstract

In today’s digital age, data centers have become one of the most important infrastructures in businesses and organizations. They store and manage critical enterprise data and resources, as well as being the core support for business operations. However, as data centers grow and expand, cybersecurity has become an important challenge. In order to efficiently retrieve ciphertexts and achieve secure communication between data center and data user, this article proposes a multi-ciphertext equality test broadcast signcryption scheme. The scheme uses broadcast signcryption technology to ensure the confidentiality and unforgeability of messages, and uses multi-ciphertext equality test to achieve efficient retrieval of ciphertexts on cloud servers. Based on the hypothesis of difficult problems, the security of the scheme has been proven under the random oracle model. Numerical analysis shows that our work has relatively high computational and communication efficiency, when the number of receivers is 100, the computational efficiency of our scheme has increased by more than 20% compared to the existing schemes and is suitable for data communication in data center.

## Introduction

**Fig 1:**
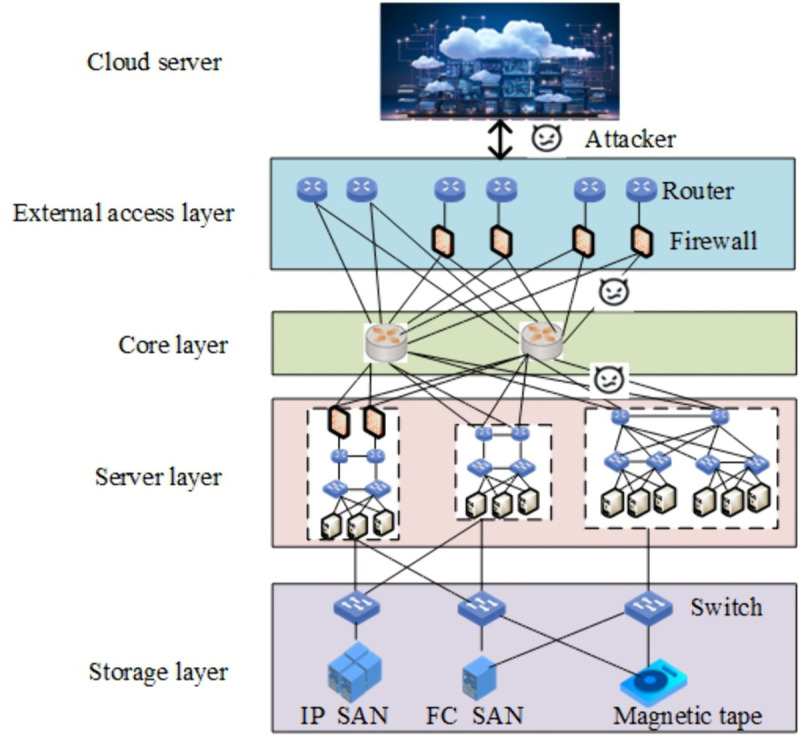
The network architecture of the data center.

**Fig 2:**
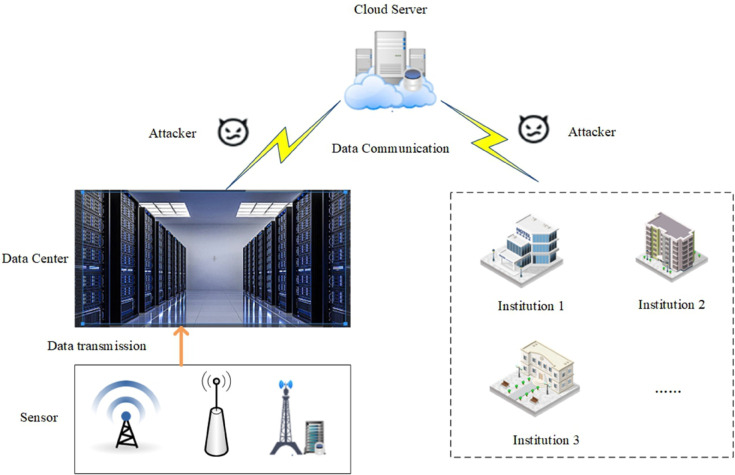
The attack of data communication.

In today’s information age, massive amounts of information flood into terminal devices every day, resulting in the problem of digital devices storing and calculating the received massive data. Data centers have become the infrastructure for storing and managing data in the digital age. The network architecture of the data center is shown in Fig 1. During the process of data transmission, data centers face many security issues such as data leakage, unauthorized access, and illegal operations. Security is the core of data center construction, and ensuring the security control of physical space and network is the cornerstone of guaranteeing the stable operation of data centers. Fig 2 shows the hacker attack paths that exist in data communication. After the sensors transmit the collected data to the data center, there may be malicious attackers attacking sensitive data in data communication.

To protect data privacy and achieve information security, data owner encrypts sensitive information and stores it in the cloud server, which poses a challenge for cloud server to perform calculations on encrypted data. To resist honest and curious cloud servers and various malicious attacks from external sources, scholars have proposed the concept of searchable encryption, the data owner only needs to encrypt the message once to achieve the function of multiple receivers searching simultaneously. However, searchable encryption only applies to ciphertext encrypted with the same public key. In order to address the limitations of searchable encryption, scholars have proposed the concept of equality test [1], which can retrieve ciphertexts encrypted with different public keys. Equality test has also been well applied in practical environments, as it is possible to retrieve ciphertexts encrypted with different public keys, and equality test has also been well applied in practical environments. Zhao et al. [2] proposed a dual server uncertified public key encryption scheme that supports authorization equality test in bilinear groups, providing confidentiality protection for outsourced ciphertexts and authentication tokens, and can resist internal keyword guessing attacks. Yuan et al. [3] proposed an image centric privacy protection architecture for social discovery services, which allows people to find friends with similar interests by testing the similarity of encrypted images. Zhao et al. [4] proposed an authorization equality test scheme for identity based cryptographic systems, which allows servers to test whether two different ciphertexts encapsulate the same message. Additionally, it supports different authorization methods to enhance privacy. However, the equality test in the above scheme only targets two ciphertexts. In order to improve the retrieval efficiency of ciphertexts, we propose a broadcast signcryption scheme with multi-ciphertext equality test in data center.

### Our contributions

– The scheme realizes data confidentiality, unforgeability and receiver’s identity anonymity. We use hybrid signcryption to ensure data security and achieve secure communication between data center and data user. The data receivers do not know each other’s identities, ensuring the anonymity of the receivers.
– Our proposed scheme carries out multi-ciphertext equality test to implement efficient retrieval of ciphertexts. When data users need to retrieve ciphertexts, they upload trapdoors to the cloud server, which performs multi-ciphertext equality test based on the trapdoors.
– Our proposed work has higher computation efficiency than existing schemes and implement a lightweight broadcast signcryption scheme. We use hybrid signcryption to ensure data security while also improving the efficiency of algorithms in the data center.

### Organization

The article is organized as follows. The Related works section summarizes the related work. The Preliminaries section introduces the preliminary work. The proposed scheme section presents the scheme structure and correctness. The Security proof section proves the security of our work. The Performance evaluation section shows the performance evaluation. Finally, we conclude this work in the Conclusion section.

## Related works

Yang et al. [1] first proposed Public Key Encryption with support for equality test(PKEET), and provided a specific scheme that anyone can check whether the messages of any two ciphertexts are the same without decrypting. In order to meet different privacy needs, Tang [5] introduces agents in the PKEET, only authorized users can perform equality test. Ma et al. [6] further limit the scope of authorization to only designated agents authorized by the user who can access the user’s confidential information, they also proposed a PKEET scheme that implemented four types of authorization strategies. Wu et al. [7] proposed an efficient identity based scheme with bilinear pairing, which reduces the time-consuming function computation and each trapdoor can only be used to perform equality test on specific keywords. However, the above scheme only compare two ciphertexts and have lower efficiency. In order to settle above problems, Susilo et al. [8] proposed an encryption scheme that supports multi ciphertext equality test, which can be used to test the relationship between multiple messages. Each ciphertext can be assigned a number *s*, allowing cloud servers to only perform equality test on that ciphertext with other *s*–1 ciphertexts. Unfortunately, the above PKEET schemes only ensure the confidentiality of information, but cannot guarantee unforgeability of data. In order to achieve both confidentiality and unforgeability of sensitive data within a single time unit, scholars have proposed a signcryption scheme [9–11]. For the reason that settle various hacker attacks and guarantee data security in different cryptosystems communication, heterogeneous signcryption scheme [12–14] have been proposed to meet sensitive information’s confidentiality and unforgeability during data transmission. However, the above works suitable for one to one transmission environment. To meet the requirements of one to many communication, Yu et al. [15] proposed a provably secure multi receiver implicit certificate based signcryption scheme based on implicit certificate cryptosystem and polynomial interpolation evaluation for one to many communication in edge computing. Wang et al. [16] proposed a secure certificate free multi receiver signature scheme to address the security issues of remote downlink control command multicast in advanced metering infrastructure. This scheme not only ensures the confidentiality, integrity, and unforgeability of commands sent by power companies, but also prevents the identity of smart meters receiving commands from being leaked. In order to improve the computational efficiency of the scheme. Shen et al. [17] proposed a lightweight uncertified data secure transmission protocol in wireless body area network environments. Zhang et al. [18] proposed a multi-receiver conditional anonymous signcryption scheme in the internet of vehicles, the scheme use attributed-based and signcryption achieve security of sensitive data. Zhang et al. [19] utilized signcryption and blockchain designed secure generic communication framework. Zhu et al. [20] used lattice-based and proxy signature proposed a proxy signcryption scheme, which have strong identifiability and defeat quantum attack. Niu et al. [21] proposed broadcast signcryption scheme to addressed the data security issues in wireless sensor network.Tanveer et al. [22] have presented a chaotic map-based authenticated data sharing framework for the IoT-enabled cloud storage environment. This scheme uses chaotic map, hash functions and encryption algorithm to ensure the confidentiality, integrity and authentication of data. Tanveer et al. [23] have presented an access control scheme based on an authenticated encryption algorithm. The schemes use an authenticated encryption algorithm, called elliptic curve cryptography, and hash function to achieve data sharing. However, above schemes do not implement the retrieval function for ciphertext. Table 1 summarizes some literatures mentioned above. In order to guarantee sensitive information security while also achieve multi-ciphertext retrieval, we propose the broadcast signcryption scheme used certificateless cryptography and multi-ciphertext equality test.

**Table 1:** Summarized literature.

References	Shortcoming	Advantage
Ref [1]	cannot guarantee the unforgeability of the message	ciphertext classification
Ref [2]	certificate management issues	resist internal keyword guessing attacks
Ref [3]	cannot guarantee the unforgeability of the message	privacy protection
Ref [4]	key escrow	authorization
Ref [5]	unable adapt to one-to-many communication scenarios	authorization
Ref [6]	certificate management issues	privacy protection, authorization
Ref [7]	key escrow	lightweight scheme
Ref [8]	cannot guarantee the unforgeability of the message	ensure the confidentiality of information
Ref [9]	simultaneously ensuring the confidentiality and unforgeability of the message	avoiding certificate management issue
Ref [10]	key escrow	lightweight scheme
Ref [11]	unable adapt to one-to-many communication scenarios	avoiding certificate management issues
Ref [12]	unable adapt to one-to-many communication	adapt to heterogeneous communication
Ref [13]	unable adapt to one-to-many communication	ciphertext classification
Ref [14]	key escrow	adapt to heterogeneous communication
Ref [15]	no equality test function	adapt to one-to-many communication
Ref [16]	cannot search and classify ciphertext	adapt to one-to-many communication
Ref [17]	cannot search and classify ciphertext	adapt to one-to-many communication
Ref [18]	cannot search and classify ciphertext	anonymous
Ref [19]	cannot search and classify ciphertext	adapt to one-to-many communication
Ref [20]	key escrow	lightweight scheme
Ref [21]	Cannot search and classify ciphertext	adapt to one-to-many communication
Ref [24]	key escrow	ciphertext classification
Ref [22]	cannot guarantee the unforgeability of the message	data access control
Ref [23]	cannot guarantee the unforgeability of the message	authorization

## Preliminaries

### System model

**Fig 3:**
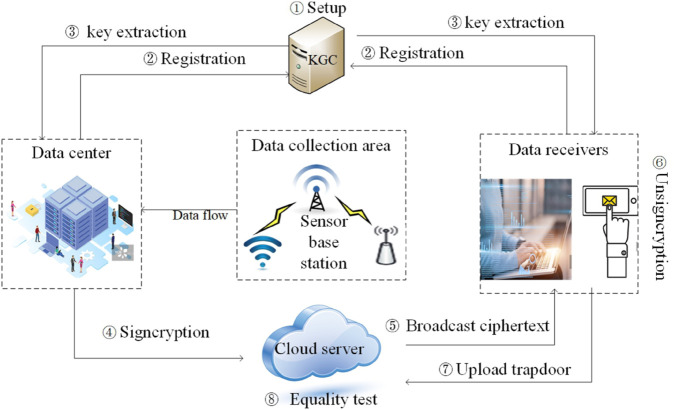
The system model of scheme.

The scheme defines four entities: Key Generation Center (KGC), data center, cloud server, and data receiver. Firstly, KGC initializes the system and generates public and partial private keys for data center and receivers. Then, the data center signcrypts the data information, generates ciphertext, and uploads it to the cloud server. The cloud server broadcasts the ciphertext to the data receivers. Finally, the data receiver verifies and decrypts the ciphertext. When the data receiver wants to retrieve the ciphertext, they upload a trapdoor to the cloud server, which executes an equality test algorithm to retrieve the ciphertext. The system model is shown in Fig 3. The definition of our proposed work includes the five algorithms as follows:

- Setup: KGC carries out this algorithm, inputs the security parameters *λ*, and outputs system public parameters *Pars*, retain master key *MSK*.
- Keygen: Users (data center and receivers) and KGC jointly implement the algorithm, given the user’s identities *U*, public parameters *Pars* and master key *MSK*, generate the user’s public *PK*_*i*_ and private keys *SK*_*i*_.
- Signcrypt: The data center implements this algorithm, inputs public parameters *Pars*, messages set *M*, the data center’s private key *SK*_*s*_ and receiver’s public key *PK*_*i*_, and outputs ciphertext *δ*.
- Unsigncrypt: Data receiver carrys out the algorithm, given ciphertext *δ*, data center’s public key *PK*_*s*_ and receiver’s private key *SK*_*i*_. Receiver checks whether the ciphertext is valid. If the message is unforgeable, it decrypts the ciphertext with its own private key to obtain the plaintext.
- Equality test: Given λ ciphertexts, the maximum number of ciphertexts that can be equality test with each ciphertext is λ − 1. Data receiver uploads the trapdoor *tk*_*i*_ to the cloud server, which performs equality test on the ciphertexts to achieve efficient retrieval of ciphertexts.

### Security model

For polynomial adversary A, if the following game cannot be won with a non negligible probability, the proposed work meet indistinguishability under the chosen ciphertext attack (IND-CCA) security, existential unforgeability of chosen message attack (EUF-CMA) security, and anonymity indistinguishability under the chosen ciphertext attack (ANON-IND-CCA) security. Adversary A and challenger C as follows:

**Game 1. IND-CCA security**

**Setup:** C generates the system master *MSK* key and public parameters *Pars*, sends *Pars* to A, and retains *MSK*.

**Phase 1:** C selects the target identity set $\left\{ID_{1},\cdots ,ID_{n}\right\}$ and sends it to the adversary, who initiates a series of adaptive queries.

**Challenge:** A selects two challenge messages and sends them to the challenger. C selects c∈{0,1} and returns the ciphertext *δ* to A.

**Phase 2:** A initiates a series of inquiries to C as Phase 1, except uses $ID_{i}\in I{D_{1}}^{*},\cdots ,I{D_{n}}^{*}$, interact $I{D_{s}}^{*}$ and $\delta^{*}$ executes unsigncryption query.

**Guess:** A outputs $c^{\prime }\in \{0,1\}$, if $c^{\prime }=c$, the adversary wins the game. The adversary’s advantage is: Adv(A)= $\left|\mathrm{Pr}(c^{\prime }=c\right)$ − $\frac{1}{2}|$.

**Definition 1.** In polynomial time, if no adversary  calAcan win Game 1 with an undeniable advantage, then the scheme satisfies IND-CCA security.

**Game 2. EUF-CMA security**

**Setup:** C generates the system master key and system public parameters, sends the system public parameters to the A, and retains the system master key.

**Query:** A initiates a series of adaptive queries, and C responds to the queries.

**Forgery:** A outputs a forged ciphertext *CT*  of the message *m* . If *CT*  is valid, A wins Game 2.

**Definition 2.** In polynomial time, if no adversary A can win Game 2 with an undeniable advantage, then the scheme satisfies EUF-CMA security.

**Game 3. ANON-IND-CCA security**

**Setup:** C generates the system master *MSK* key and public parameters *Pars*, sends *Pars* to A, and retains *MSK*.

**Phase 1:** C selects the target identity $\left\{ID_{r},I{D_{r}}^{*}\right\}$ and sends it to A, who initiates a series of adaptive queries.

**Phase 2:** A initiates a series of inquiries to C as Phase 1, except uses $ID_{i}\in \{I{D_{1}}^{*},\cdots ,I{D_{n}}^{*}\}$, $I{D_{s}}^{*}$ and $\delta^{*}$ executes unsigncryption query.

**Challenge:** A selects multiple challenge messages and sends it to the challenger. C selects c∈{0,1} and returns the ciphertext *δ* to A.

**Guess:** A outputs $c^{\prime }\in \{0,1\}$, if $c^{\prime }=c$, the adversary wins the game. The adversary’s advantage is: Adv(A)= $\left|\mathrm{Pr}(c^{\prime }=c\right)$ − $\frac{1}{2}|$.

**Definition 3.** In polynomial time, if no adversary A can win Game 3 with an undeniable advantage, then the scheme satisfies ANON-IND-CCA security.

## The proposed scheme

This section mainly introduces the specific algorithm of the proposed scheme. In order to achieve secure communication and efficient retrieval of ciphertext, a broadcast signcryption scheme with multi-ciphertext equality test is proposed. Table 2 shows the notation of the scheme. The scheme consists of the following five algorithms:

**Table 2:** Notations.

Notations	Description
*P*	Generator of group
*λ*	Security parameter
$H,H_{1},H_{2},H_{3},H_{4},H_{5}$	Hash functions
*Pars*	System public key
$v_{i},u_{i},r$	Random numbers
*SK*_*i*_	Private key
*PK*_*i*_	Public key
*d*_*i*_	Partial private key
*M*	Message
*δ*	Ciphertext

**Setup:** This algorithm is executed by KGC. Enter the security parameter *λ*, KGC outputs the system’s public parameters, and retains the master key.

- Given a cyclic additive group *G* of prime order *p*, where G=<P> ;
- Define five hash functions: $H_{1}:\{0,1{\}}^{*}\to Z_{q}^{*}$, $H_{2}:\{0,1{\}}^{*}\times G\to Z_{q}^{*}$, $H_{3}:\{0,1{\}}^{*}\times G\times \{0,1{\}}^{*}\to Z_{q}^{*}$, $H_{4}:Z_{q}^{*}\to \psi$, $H_{5}:G\to Z_{q}^{*}$, where *ψ* is key space;
- KGC randomly selects $s\in Z_{q}^{*}$ as master key, computes $P_{pub}=s\cdot P$, outputs $Pars=\{G,H_{1},H_{2},H_{3},H_{4},H_{5},P_{pub},Enc(),Dec()\}$, retains *s*, where (*Enc*, *Dec*) are encryption and decryption algorithm.

**Keygen:** This algorithm is executed by data users and KGC. KGC generates partial private key and sends it to data users, who verify the legality of the partial private keys and calculate private key.

- Given data users set $U=\{ID_{s},ID_{1},ID_{2},\cdots ,ID_{n}\}$, each data user randomly selects $u_{i}\;\in \;Z_{q}^{*}$ as secret value, computes $U_{i}\;=\;u_{i}\cdot P$ and sends to KGC;
- KGC selects $v_{i}\in Z_{q}^{*}$, computes $V_{i}=v_{i}\cdot P$, public key $PK_{i}=H_{1}(ID_{i})\cdot U_{i}+V_{i}$, partial private key $d_{i}=v_{i}+s\cdot H_{2}(ID_{i},PK_{i})$, and sends $V_{i}$, *d*_*i*_ to data users;
- Data users verify the legitimacy of partial private keys by checking whether equation $d_{i}\cdot P=V_{i}+P_{pub}\cdot H_{2}(ID_{i},PK_{i})$ holds true. If the equation holds true, computes private key $SK_{i}=H_{1}(ID_{i})\cdot U_{i}+d_{i}\cdot P$, otherwise the algorithm is aborted.

**Signcryption:** This algorithm is executed by the data center. Given the system’s public parameters, message set, and data receiver identity, the data center outputs ciphertext.

- Inputs *Pars*, $M=\{M_{1},M_{1},\cdots ,M_{m}\}$, data center randomly selects $r\in Z_{q}^{*}$, computes R=r · P, $K_{i}=r\cdot (PK_{i}+P_{pub}\cdot H_{2}(ID_{i},PK_{i}))$, $\xi_{i}=H_{3}(ID_{i},R,K_{i})$;
- Rnomly selects $(t_{1},t_{2},\cdots ,t_{m})\in Z_{q}^{m}$, computes

$\left(\begin{array}{c} w_{o}\\ w_{1}\\ \;\vdots \\ w_{n-1} \end{array}\right)=(\begin{array}{cccc} 1 & \xi_{1} & \cdots & {\xi_{1}}^{n-1}\\ 1 & \xi_{2} & \cdots & {\xi_{2}}^{n-1}\\ \vdots & \vdots & \cdots & \vdots \\ 1 & \xi_{n} & \cdots & {\xi_{n}}^{n-1} \end{array}{)}^{-1}\left(\begin{array}{c} t_{\Gamma [1]}\\ t_{\Gamma [2]}\\ \;\vdots \\ t_{\Gamma [m]} \end{array}\right)$

- Computes $\varphi (x)=w_{0}+w_{1}x+\cdots +w_{n-1}x^{n-1}$, sets $W=\{w_{0},w_{1},\cdots ,w_{n-1}\in Z_{q}^{n}\}$, where Γ is a mapping table from [1,m] to [1,*n*], computes key $k_{j}=H_{4}(t_{j})$, where j∈[1.m], ciphertext $C_{j}=Enc(k_{j},M_{j})$, *CT* = {*W*,*C*_*j*_}, $h=H_{3}(ID_{s},R,CT)$, $A=R-h\cdot SK_{s}$;
- Given that the number of ciphertexts for equality test with each ciphertext is *l*, computes coefficients $f_{j,0}=H_{1}(M_{j}||l)$, $f_{j,1}\;=\;H_{1}(M_{j}||l||f_{j,0})$, ⋯, $f_{j,l-1}=H_{1}(M_{j}||l||f_{j,l-2})$, generates polynomial $f(y)=f_{j,0}+f_{j,1}y+\cdots +f_{j,l-1}y^{l-1}$, randomly selects $b_{j}\in Z_{q}^{*}$, computes $C_{j,1}=H_{5}(V_{i}+P_{pub}\cdot H_{2}(ID_{i},PK_{i}))⊕(b_{j}||f(b_{j}))$, $C_{j,2}=l||C_{j}||C_{j,1}||f_{0}||\cdots ||f_{l-1}$, outputs ciphertext $\delta =\{CT,h,C_{j,1},C_{j,2}\}$.

**Unsigncryption:** This algorithm is executed by the data receiver. After receiving the broadcast ciphertext from the cloud server, the data receiver decrypts ciphertext using the private key and verifies the unforgeability using data center’s public key.

- Inputs receiver’s *SK*_*i*_ and data center’s *PK*_*s*_, receiver computes $R^{\prime }=A+h\cdot (PK_{s}+P_{pub}\cdot H_{2}(ID_{s},PK_{s}))$, $h^{\prime }=H_{3}(ID_{s},R^{\prime },CT)$, and checks $h^{\prime }=h$ whether holds;
- If equation holds, computes ${K_{i}}^{\prime }=SK_{i}$ ⋅ $R^{\prime }$, ${\xi_{i}}^{\prime }=H_{3}(ID_{i},R^{\prime },{K_{i}}^{\prime })$, ${t_{j}}^{\prime }=\varphi ({\xi_{i}}^{\prime })$, ${k_{j}}^{\prime }=H_{4}({t_{j}}^{\prime })$, decrypts and obtains message ${M_{j}}^{\prime }=Dec({k_{j}}^{\prime })$.

**Equality-test:** This algorithm is executed by cloud server. The cloud server performs multi-ciphertext equality test based on the trapdoor of receivers to achieve the function of retrieving ciphertext.

- Given *λ* ciphertexts, the maximum number of ciphertext that can be equality test with each ciphertext is λ−1, receiver uploads trapdoor $tk_{i}=d_{i}$ to cloud server, cloud server computes $b_{j}||f(b_{j})=C_{j,1}⊕H_{5}(d_{i}\cdot P)$;
- From $f(b_{j})=f_{j,0}+f_{j,1}b_{j}+\cdots +f_{j,l-1}{b_{j}}^{l-1}$ can be obtain

$\left\{ \begin{array}{c} f(b_{1})=f_{1,0}+f_{1,1}b_{1}+\cdots +f_{1,\lambda -1}{b_{1}}^{\lambda -1}\\ f(b_{2})=f_{2,0}+f_{2,1}b_{2}+\cdots +f_{2,\lambda -1}{b_{2}}^{\lambda -1}\\ \;\vdots \\ f(b_{\lambda })=f_{\lambda ,0}+f_{\lambda ,1}b_{\lambda }+\cdots +f_{\lambda ,\lambda -1}{b_{\lambda }}^{l-1} \end{array}\right.$

- Set $f_{i,k}=f_{j,k}$, where i,j∈{1,2,⋯,λ}, k∈{0,1,⋯, λ−1}, the system of equations has a unique solution $f_{1,0},f_{1,1},\cdots ,f_{1,\lambda -1}$;
- Checks equation $C_{j,2}=\lambda ||C_{j}||C_{j,1}||f_{j,0}||\cdots ||f_{j,\lambda -1}$ whether holds,if holds,outputs 1, otherwise, outputs 0.

**Correctness:**

(1) To verify the legality of partial private keys, it is necessary to prove the following equation holds true:

$d_{i}\cdot P=v_{i}\cdot P+s\cdot P\cdot H_{2}(ID_{i},PK_{i})\;=V_{i}+P_{pub}\cdot H_{2}(ID_{i},PK_{i})$

(2) To prove the correctness of the signature, it is necessary to verify the following equation holds true:

$R^{\prime }=A+h\cdot (PK_{s}+P_{pub}\cdot H_{2}(ID_{s},PK_{s}))\;=R-h\cdot H_{1}(ID_{s})\cdot U_{s}+h\cdot d_{s}\cdot P+h\cdot H_{1}(ID_{s})\;\cdot U_{s}+h\cdot P_{pub}\cdot H_{2}(ID_{s},PK_{s})\;=R$

(3) By the following calculation get $t_{j}^{\prime }$:

$\left(\begin{array}{c} t_{\Gamma [1]}\\ t_{\Gamma [2]}\\ \;\vdots \\ t_{\Gamma [n]} \end{array}\right)=\left(\begin{array}{cccc} 1 & \xi_{1} & \cdots & {\xi_{1}}^{n-1}\\ 1 & \xi_{2} & \cdots & {\xi_{2}}^{n-1}\\ \vdots & \vdots & \cdots & \vdots \\ 1 & \xi_{n} & \cdots & {\xi_{n}}^{n-1} \end{array}\right)\left(\begin{array}{c} w_{o}\\ w_{1}\\ \;\vdots \\ w_{n-1} \end{array}\right)$

From $t_{j}^{\prime }$ compute ${k_{j}}^{\prime }=H_{4}({t_{j}}^{\prime })$, obtain message ${M_{j}}^{\prime }=Dec({k_{j}}^{\prime })$.

(4) To prove the correctness of the equality test, it is necessary to verify that the following system of equations has a unique solution:

$V=\left(\begin{array}{cccc} 1 & {\text{b}}_{1} & \cdots & {{\text{b}}_{1}}^{\lambda -1}\\ 1 & {\text{b}}_{2} & \cdots & {{\text{b}}_{2}}^{\lambda -1}\\ \vdots & \vdots & \cdots & \vdots \\ 1 & {\text{b}}_{\lambda } & \cdots & {{\text{b}}_{\lambda }}^{\lambda -1} \end{array}\right)$

$\det(V)=\prod_{1\leq i\leq j\leq \lambda }(b_{i}-b_{j})\neq 0$, so the system of equation has unique solution, $f_{i,k}=f_{j,k}$ hold.

## Security proof

Define two types of adversaries: $A_{I}$ and $A_{II}$, where adversary $A_{I}$ is a malicious user who can replace the user’s public key but cannot access the master key, and adversary $A_{II}$ is a malicious KGC who is allowed to access the system’s master key but cannot replace the user’s public key. The scheme has been proven to be safe under the random oracle model.

**Theorem 1.** If no $A_{I}$ wins Game 1 with non-negligible advantage, then the scheme has IND-CCA security.

**Proof.** $A_{I}$ and C interact as follows:

**Setup:** C sets $\left(s,P_{pub})=(⊥,P_{pub}\right)$, and sends public parameters *Pars* to $A_{I}$.

**Phase 1:** $A_{I}$ selects sender’s identity $I{D_{s}}^{*}$, *n* receiver’s identities $R^{*}=\left\{I{D_{1}}^{*},\cdots ,I{D_{n}}^{*}\right\}$ and send to C, C creates a empty list *L*_*i*_ and $A_{I}$ interact as follows:

***H***_**1**_ **query:** Given *ID*_*i*_, challengr C checks list *L*_1_ whether exist $\left(ID_{i},U_{i},V_{i},H_{1}(ID_{i})\right)$, if exist, returns $H_{1}(ID_{i})$, otherwise, randomly selects $H_{1}(ID_{i})$ and stores tuple $\left(ID_{i},U_{i},V_{i},H_{1}(ID_{i})\right)$ in *L*_1_;

***H***_**2**_ **query:** Given $\left(ID_{i},PK_{i}\right)$, challenger C checks list *L*_2_ whether exist $(ID_{i},PK_{i},H_{2}($

$ID_{i},PK_{i}))$, if exist, C returns $H_{2}(ID_{i},PK_{i})$, otherwise, C randomly selects $H_{2}(ID_{i},PK_{i})$, stores $\left(ID_{i},PK_{i},H_{2}(ID_{i},PK_{i})\right)$ in *L*_2_;

***H***_**3**_ **query:** Given $\left(ID_{i},R,K_{i}\right)$, C checks list *L*_3_ whether exist $\left(ID_{i},R,K_{i},\xi_{i}\right)$, if exist, returns $\xi_{i}$, otherwise, randomly selects $\xi_{i}$, stores $\left(ID_{i},R,K_{i},\xi_{i}\right)$ in list *L*_3_;

**Key query:** Given *ID*_*i*_, C checks list *L*_*U*_ whther exist $\left(ID_{i},u_{i},v_{i},U_{i},V_{i},SK_{i},PK_{i}\right)$, if exist, returns public key *PK*_*i*_ to $A_{I}$, otherwise, randomly selects $u_{i},v_{i}\in Z_{p}^{*}$, computes $U_{i}=u_{i}\cdot P$, $V_{i}=v_{i}\cdot P$, $PK_{i}=H_{1}(ID_{i})\cdot U_{i}+V_{i}$, sets $SK_{i}=⊥$ and updates *L*_*U*_, sends *PK*_*i*_ to $A_{I}$.

**Secret-value query:** Given *ID*_*i*_, C checks list *L*_*U*_ whether exist $\left(ID_{i},u_{i},v_{i},U_{i},V_{i},SK_{i},PK_{i}\right)$, if $ID_{i}\in R^{*}$, returns ⊥, otherwise, outputs $\left(u_{i},U_{i}\right)$.

**Public key replace query:** Input $\left(ID_{i},P{K_{i}}^{\prime }\right)$, C checks list *L*_*U*_ whether exist $\left(ID_{i},u_{i},v_{i},U_{i},V_{i},SK_{i},PK_{i}\right)$, if exist, C uses $P{K_{i}}^{\prime }$ repalces *PK*_*i*_, otherwise, uses *ID*_*i*_ initiates public key replace query.

**Signcryption query:** Given identites set $\left\{ID_{s},ID_{1},\cdots ,ID_{n}\right\}$, message $\left\{M_{1},\cdots ,M_{n}\right\}$, if $sk_{i}=⊥$, challenger C randomly selects $r,s,{\text{t}}_{1},\cdots ,\;{\text{t}}_{m}\in Z_{p}^{*}$, computes $K=s\cdot P+r\left(PK_{s}+P_{pub}\cdot H_{2}(ID_{i},PK_{i})\right)$, $C_{i}=Enc(H_{4}(t_{i}),M_{i})$, stores $\left(ID_{i},⊥,K,t_{i}\right)$ and $\left(ID_{s},K,C_{i},r\right)$ in *L*_3_, otherwise, C uses $\left(PK_{1},\cdots ,PK_{n}\right)$ executes signcryption algorithm, generates ciphertext $\delta =\{CT,h,a,C_{j,1},C_{j,2}\}$ and sends to adversary $A_{I}$.

**Unsigncryption query:** Given *ID*_*s*_, *ID*_*i*_, *δ*, challenger C computes *K* = *s* ⋅ *P*  +  $r\left(PK_{s}+P_{pub}\cdot H_{2}(ID_{i},PK_{i})\right)$, searches by (*ID*_*i*_,*K*) for $\left(ID_{s},K,C_{i},r\right)$, computes ${M_{j}}^{\prime }=Dec(H_{4}(t_{j}))$ to adversary $A_{I}$.

**Challenge:** $A_{I}$ selects two message sets of equal length $M=\{M_{1},\cdots ,M_{m}\}$, $M^{*}=\{{M_{1}}^{*},\cdots ,{M_{m}}^{*}\}$ and sends to C , C randomly selects c∈{0,1}, $r,s,\;{\text{t}}_{1},\cdots ,\;{\text{t}}_{m}\in Z_{p}^{*}$, computes $K=s\cdot P+r\left(PK_{s}+P_{pub}\cdot H_{2}(ID_{i},PK_{i})\right)$, $C_{i}=Enc(H_{4}(t_{i}),M_{i})$, outputs challenge ciphertext $\delta^{*}=\{CT^{*},h^{*},{C_{j,1}}^{*},{C_{j,2}}^{*}\}$ to adversary $A_{I}$.

**Phase 2:** $A_{I}$ initiates the same query to C as phase 1, but can’t uses $ID_{i}\in \{I{D_{1}}^{*},\cdots ,I{D_{n}}^{*}\}$, $I{D_{s}}^{*}$ and $\delta^{*}$ executes unsigncryption algorithm.

**Guess:** $A_{I}$ outputs the guess result $c^{\prime }\in \{0,1\}$, if $c^{\prime }=c$, adversary $A_{I}$ wins the game. If $A_{I}$ wins the game with non-negligible advantage, must have the right *t*_*i*_, and uses $\left(ID_{i},PK_{i}\right)$ initiates *H*_2_ query to make $K_{i}=r\cdot (PK_{i}+P_{pub}\cdot H_{2}(ID_{i},PK_{i}))=r\cdot (PK_{i}+s\cdot P\cdot H_{2}(ID_{i},PK_{i}))$. $rsP=(K_{i}-r\cdot PK_{i})\cdot {H_{2}}^{-1}(ID_{i},PK_{i})$ as a solution to the CDH difficulty problem, the probability of $A_{I}$ wins Game 1 is: $Adv(A_{I})=|\mathrm{Pr}(c^{\prime }=c)-\frac{1}{2}|$.

**Theorem 2.** If no $A_{II}$ wins Game 1 with non-negligible advantage, then the scheme has IND-CCA security.

**Proof.** $A_{II}$ and C interact as follows:

**Setup:** C sets $\left(s,P_{pub})=(⊥,P_{pub}\right)$, sends public parameters *Pars* to $A_{II}$.

**Phase 1:** It is the same as Theorem 1 except public key repalce query. Given *ID*_*i*_, C checks list *L*_*U*_ whether exist $\left(ID_{i},u_{i},v_{i},U_{i},V_{i},SK_{i},PK_{i}\right)$, if exist, returns *PK*_*i*_ to $A_{II}$, otherwise, randomly selects $u_{i},v_{i}\in Z_{p}^{*}$, computes $U_{i}=u_{i}\cdot P$, $V_{i}=v_{i}\cdot P$, $PK_{i}=H_{1}(ID_{i})\cdot U_{i}+V_{i}$, sets $SK_{i}=⊥$, and updates *L*_*U*_, sends *PK*_*i*_ to $A_{II}$.

**Phase 2:** $A_{II}$ initiates the same query to C as phase 1, but can’t uses $ID_{i}\in \{I{D_{1}}^{*},\cdots ,I{D_{n}}^{*}\}$, $I{D_{s}}^{*}$ and $\delta^{*}$ executes unsigncryption algorithm.

**Guess:** $A_{II}$ outputs the guess result $c^{\prime }\in \{0,1\}$, if $c^{\prime }=c$, $A_{II}$ wins the game. If adversary $A_{II}$ wins the game with non-negligible advantage, must have the right *t*_*i*_ and uses $\left(ID_{i},PK_{i}\right)$ initiates *H*_2_ query to make $K_{i}=r\cdot (PK_{i}+P_{pub}\cdot H_{2}(ID_{i},PK_{i}))=r\cdot (PK_{i}+s\cdot P\cdot H_{2}(ID_{i},PK_{i}))$. $rsP=(K_{i}-r\cdot PK_{i})\cdot {H_{2}}^{-1}(ID_{i},PK_{i})$ as a solution to the CDH difficulty problem, the probability of $A_{II}$ wins Game 1 is: $Adv(A_{II})=|\mathrm{Pr}(c^{\prime }=c)-\frac{1}{2}|$.

**Theorem 3.** If no adversary $A_{I}$ wins Game 2 with non-negligible advantage,the scheme satisfies the EUF-CMA security.

**Proof.** C and $A_{I}$ interact as follows:

**Setup:** C sets master key $\left(s,P_{pub})=(⊥,Q\right)$, and sends public parameters *Pars* to $A_{I}$.

**Phase 1:** $A_{I}$ can adaptively initiate queries, similar to phase 1 and 2 in Theorem 1, excepts key query and secret-value query.

**key query:** Given identity *ID*_*i*_, challenegr C checks whether $\left(ID_{i},u_{i},v_{i},U_{i},V_{i},SK_{i},PK_{i}\right)$ exist in *L*_*U*_. If exist, C returns *PK*_*i*_ to $A_{I}$, otherwise, C randomly selects $u_{i},v_{i}\in Z_{p}^{*}$, computes $U_{i}=u_{i}\cdot P$, $V_{i}=v_{i}\cdot P$, $PK_{i}=H_{1}(ID_{i})\cdot U_{i}+V_{i}$, sets $SK_{i}=⊥$ and updates *L*_*U*_, sends *PK*_*i*_ to $A_{I}$.

**secrect-value query:** Given *ID*_*i*_, C checks whther *L*_*U*_ exist in $\left(ID_{i},u_{i},v_{i},U_{i},V_{i},SK_{i},PK_{i}\right)$, if exist, sets $SK_{i}=⊥$, C returns $\left(SK_{i},PK_{i}\right)$ to $A_{I}$. Otherwise, C randomly selects $u_{i},v_{i},d_{i}\in Z_{p}^{*}$, computes $U_{i}=u_{i}\cdot P$, $V_{i}=v_{i}\cdot P$, $PK_{i}=H_{1}(ID_{i})\cdot U_{i}+V_{i}$, $SK_{i}=H_{1}(ID_{i})\cdot U_{i}+d_{i}\cdot P$, updates *L*_1_ and *L*_*U*_, sends $\left(SK_{i},PK_{i}\right)$ to $A_{I}$.

**forge:** Adversary $A_{I}$ outputs forge ciphertext $\delta^{*}=\{CT^{*},h^{*},{C_{j,1}}^{*},{C_{j,2}}^{*}\}$. If a key query with the same identity is queried first, subsequent key queries with the same identity will change the corresponding public key due to changes in the secret value generated by the user. Therefore, the ciphertext forged by the adversary $A_{I}$ is invalid.

**Theorem 4.** If no adversary $A_{II}$ wins Game 2 with non-negligible advantage, the scheme satisfies the EUF-CMA security.

**Proof.** C and $A_{II}$ interact as follows:

**Setup:** C sets master key $\left(s,P_{pub})=(⊥,Q\right)$, sends *Pars* to $A_{II}$.

**Phase 1:** $A_{II}$ can adaptively initiate queries, similar to phase 1 and 2 in Theorem 2, excepts key query and secret-value query.

**key query:** Given identity *ID*_*i*_, challenger C checks whether $\left(ID_{i},u_{i},v_{i},U_{i},V_{i},SK_{i},PK_{i}\right)$ exist in *L*_*U*_. if exist, C returns *PK*_*i*_ to $A_{II}$, otherwise, C randomly selects $u_{i},v_{i}\in Z_{p}^{*}$, computes $U_{i}=u_{i}\cdot P$, $V_{i}=v_{i}\cdot P$, $PK_{i}=H_{1}(ID_{i})\cdot U_{i}+V_{i}$, sets $SK_{i}=⊥$ and updates *L*_*U*_, sends *PK*_*i*_ to $A_{II}$.

**secret-value query:** Given *ID*_*i*_, C checks whether *L*_*U*_ exist in $\left(ID_{i},u_{i},v_{i},U_{i},V_{i},SK_{i},PK_{i}\right)$. if exist, sets $SK_{i}=⊥$, C returns $\left(SK_{i},PK_{i}\right)$ to $A_{I}$, otherwise, C randomly selects $u_{i},v_{i},d_{i}\in Z_{p}^{*}$, computes $U_{i}=u_{i}\cdot P$, $V_{i}=v_{i}\cdot P$, $PK_{i}=H_{1}(ID_{i})\cdot U_{i}+V_{i}$, $SK_{i}=H_{1}(ID_{i})\cdot U_{i}+d_{i}\cdot P$, updates *L*_1_ and *L*_*U*_, sends $\left(SK_{i},PK_{i}\right)$ to $A_{II}$.

**Forge:** Adversary $A_{II}$ outputs a forge ciphertext $\delta^{*}=\{CT^{*},h^{*},{C_{j,1}}^{*},{C_{j,2}}^{*}\}$. If a key query with the same identity is queried first, subsequent key queries with the same identity will change the corresponding public key due to changes in the secret value generated by the user. Therefore, the ciphertext forged by the adversary $A_{II}$ is invalid.

**Theorem 5.** If no adversary $A_{I}$ wins Game 3 with non-negligible advantage, the scheme satisfies the ANON-IND-CCA security.

**Proof.** Given a CDH difficulty problem, $A_{I}$ and C interact as follows:

**Phase 1:** C randomly selects $\left\{ID_{r},I{D_{r}}^{*}\right\}$ and sends to $A_{I}$, $A_{I}$ initiates the same query as theorem 1.

**Phase 2:** $A_{I}$ initiates the same query as phase 1, but can’t uses $ID_{i}\in \{I{D_{1}}^{*},\cdots ,I{D_{n}}^{*}\}$, $I{D_{s}}^{*}$ and $\delta^{*}$ initiates unsigncryption query. C randomly selects $u_{i},v_{i}\in Z_{p}^{*}$, computes $U_{i}=u_{i}\cdot P$, $V_{i}=v_{i}\cdot P$, $PK_{i}=H_{1}(ID_{i})\cdot U_{i}+V_{i}$, sets $SK_{i}=⊥$ and updates *L*_1_, *L*_*U*_, then sends *PK*_*i*_ to $A_{I}$.

**Challenge:** $A_{I}$ selects *m* messages $M=\{M_{1},\cdots ,M_{m}\}$, *n* identities $\left\{ID_{2},\cdots ,ID_{n},ID_{s}\right\}$ and sends to C. If $I{D_{s}}^{*}\neq ID_{s}$, challenger returns ⊥, otherwise, randomly selects c∈{0,1}, $r,s,{\text{t}}_{1},\cdots ,\;{\text{t}}_{m}\in Z_{p}^{*}\in Z_{p}^{*}$, sets $ID_{s}=I{D_{s}}^{c}$, and computes *K* = *s* ⋅ $P+r(PK_{s}+P_{pub}\cdot H_{2}(ID_{i},PK_{i}))$, $C_{i}=Enc(H_{4}(t_{i}),M_{i})$. Generates challenge ciphertext $\delta^{*}=\{CT^{*},h^{*},{C_{j,1}}^{*},{C_{j,2}}^{*}\}$ and sends to $A_{I}$.

**Guess:** $A_{I}$ outputs the result of guess $c^{\prime }\in \{0,1\}$. If $c^{\prime }=c$, $A_{I}$ wins the game, if adversary wins the game with non-negligible advantage, must have *t*_*i*_ and uses $\left(ID_{i},PK_{i}\right)$ initiates *H*_2_ query to make $K_{i}=r\cdot (PK_{i}+P_{pub}\cdot H_{2}(ID_{i},PK_{i}))=r\cdot (PK_{i}+s\cdot P\cdot H_{2}(ID_{i},PK_{i}))$. Outputs $rsP=(K_{i}-r\cdot PK_{i})\cdot {H_{2}}^{-1}(ID_{i},PK_{i})$ as a solution to the CDH problem the probability of $A_{I}$ wins Game 3 is: $Adv(A_{I})=|\mathrm{Pr}(c^{\prime }=c)-\frac{1}{2}|$.

**Theorem 6.** If no adversary $A_{II}$ wins Game 3 with non-negligible advantage,the scheme satisfies the ANON-IND-CCA security.

**Proof.** Given a CDH difficulty problem, $A_{II}$ and C interact as follows:

**Phase 1:** C randomly selects $\left\{ID_{r},I{D_{r}}^{*}\right\}$ and sends to $A_{I}$, $A_{I}$ initiates the same query as theorem 2.

**Phase 2:** $A_{II}$ initiates the same query as phase 1, but can’t uses $ID_{i}\in \{I{D_{1}}^{*},\cdots ,I{D_{n}}^{*}\}$, $I{D_{s}}^{*}$ and $\delta^{*}$ initiates unsigncryption query. C randomly selects $u_{i},v_{i}\in Z_{p}^{*}$, computes $U_{i}=u_{i}\cdot P$, $V_{i}=v_{i}\cdot P$, $PK_{i}=H_{1}(ID_{i})\cdot U_{i}+V_{i}$, sets $SK_{i}=⊥$ and updates *L*_1_, *L*_*U*_, sends *PK*_*i*_ to $A_{II}$.

**Challenge:** $A_{II}$ selects *m* messages $M=\{M_{1},\cdots ,M_{m}\}$, *n* identities $\left\{ID_{2},\cdots ,ID_{n},ID_{s}\right\}$ and sends to C. If $I{D_{s}}^{*}\neq ID_{s}$, challenger returns ⊥. Otherwise, C randomly selects c∈{0,1}, $r,s,{\text{t}}_{1},\cdots ,\;{\text{t}}_{m}\in Z_{p}^{*}$ and sets $ID_{s}=I{D_{s}}^{c}$, computes $K=s\cdot P+r(PK_{s}+P_{pub}\cdot H_{2}(ID_{i},PK_{i}))$, $C_{i}=Enc(H_{4}(t_{i}),M_{i})$. C generates challenge ciphertext $\delta^{*}=\{CT^{*},h^{*},{C_{j,1}}^{*},{C_{j,2}}^{*}\}$ and sends to $A_{II}$.

**Guess:** $A_{II}$ outputs the result of guess $c^{\prime }\in \{0,1\}$. If $c^{\prime }=c$, $A_{II}$ wins the game, if adversary wins the game with non-negligible advantage, must have *t*_*i*_ and uses $\left(ID_{i},PK_{i}\right)$ initiates *H*_2_ query to make $K_{i}=r\cdot (PK_{i}+P_{pub}\cdot H_{2}(ID_{i},PK_{i}))=r\cdot (PK_{i}+s\cdot P\cdot H_{2}(ID_{i},PK_{i}))$. Outputs $rsP=(K_{i}-r\cdot PK_{i})\cdot {H_{2}}^{-1}(ID_{i},PK_{i})$ as a solution to the CDH problem the probability of $A_{II}$ wins Game 3 is: $Adv(A_{II})=|\mathrm{Pr}(c^{\prime }=c)-\frac{1}{2}|$.

## Performance evaluation

We compared the functionality and analyzed the computational efficiency of the proposed scheme in this paper with the exisiting work. Numerical analysis was conducted on the execution time of algorithms in each stage of the scheme on the Linux operating system using PBC library [62] and C language.

### Functional Comparison

Table 3 compares the functional differences between existing signcryption schemes and the scheme proposed in this paper. Reference [12] proposes a heterogeneous signcryption scheme for wireless body area networks in a heterogeneous environment from IBC to PKI, which addressed the security issues in data transmission. However, the scheme is not suitable for one to many broadcasting environments. Although reference [15] achieves one to many broadcast communication security, this scheme does not support ciphertext retrieval. Reference[24] implements classification management of ciphertexts using equality test, but only compares two ciphertexts, which is inefficient, and the scheme has the problem of key leakage. We proposed the broadcast signcryption scheme achieves multi-ciphertext retrieval functions while ensuring confidentiality and unforgeability during data transmission, making it suitable for data centers.

**Table 3:** Functional comparison.

Scheme	Ref [12]	Ref [15]	Ref [22]	Ours
Key escrow	Yes	No	Yes	No
Hybrid signcryption	No	No	No	Yes
Equality test	Yes	No	Yes	Yes
Broadcast	No	Yes	No	Yes

### Experimental analysis

We compared the efficiency of the existing scheme with the scheme proposed in this paper, and our experiment is run under a Linux operation system using a pairing-based cryptography library with Type-A bilinear pairing parameters.

Table 4 shows the definition represented by each character. The computation overhead of signcryption, unsigncryption and equality test as shown in Table 5. Table 6 presents the key and ciphertext memory space of schemes. The computation and communication costs increase with the increase of the number of receivers.

**Table 4:** Character definition.

Character	Definition
*T*_*p*_	The time of bilinear operations
*T*_*e*_	The time of exponential operations
*T*_*m*_	The time of multiple operations
*T*_*p*_	The time of hash operations
$\left|Z_{p}^{*}\right|$	The length of $Z_{p}^{*}$
|G|	The length of group *G*

**Table 5:** Computation costs.

Scheme	Ref [12]	Ref [15]	Ref [22]	Ours
Signcryption	$(2n)T_{e}+3nT_{m}$ + 3*nT*_*h*_	(3*n* + 1)*T*_*m*_ + 4*nT*_*h*_	$2nT_{p}+2nT_{e}$ + 5*T*_*m*_	(2*n* + 2)*T*_*m*_ + 3*nT*_*h*_
Unsigncryption	$(2n+1)T_{p}+T_{e}$ $+nT_{m}+(2n+1)T_{h}$	(2*n* + 2)*T*_*m*_ + (2*n* + 1)*T*_*h*_	$(2n+3)T_{p}+3T_{m}$	(2*n* + 1)*T*_*m*_ + 2*nT*_*h*_
Equality test	$2nT_{p}+2nT_{e}+2nT_{m}$	–	4*nTp*	$nT_{m}+nT_{h}$

**Table 6:** Communication costs.

Scheme	Key size	Ciphertext size
Ref [12]	5n|G|	$4n\left|G\right|+\left(2n\right)\left|Z_{p}^{*}\right|$
Ref [15]	$n\left|G\right|+n\left|Z_{p}^{*}\right|$	$4n+1\left|Z_{p}^{*}\right|$
Ref [22]	3n|G|	$\left(2+n\right)\left|G\right|+2n\left|Z_{p}^{*}\right|$
Ours	2n|G|	$\left(n\right)\left|G\right|+3n\left|Z_{p}^{*}\right|$

**Fig 4:**
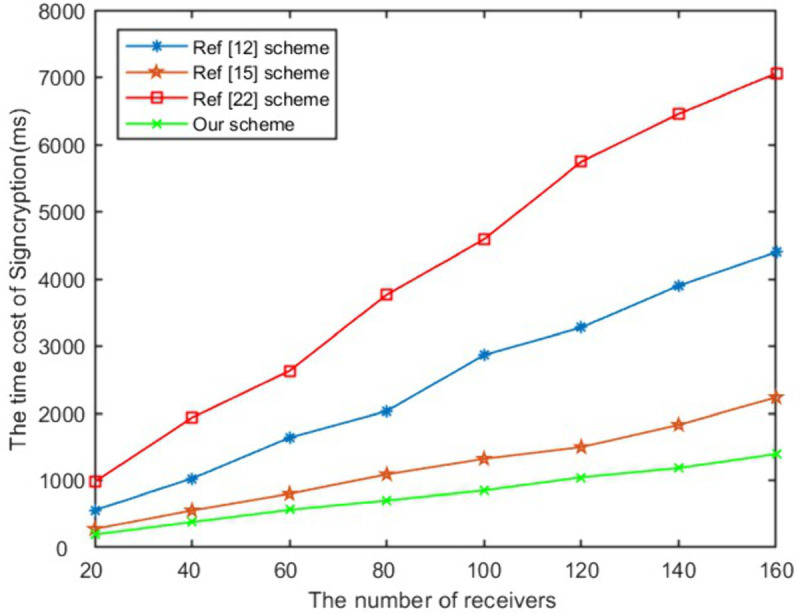
The time of signcryption.

**Fig 5:**
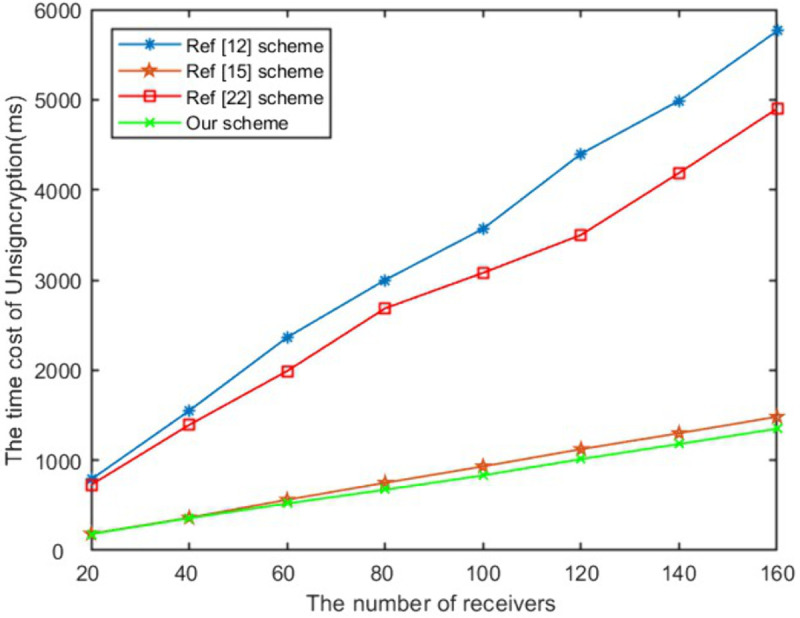
The time of unsigncryption.

**Fig 6:**
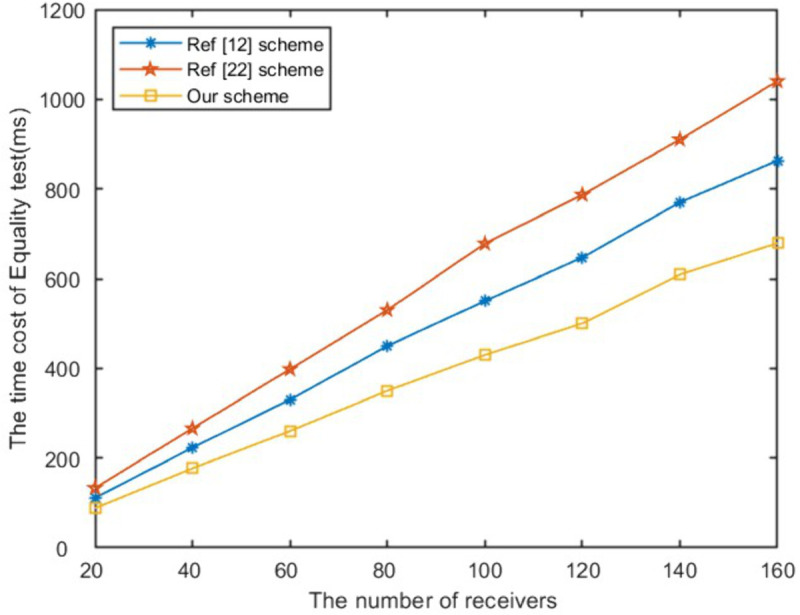
The time of equality test.

**Fig 7:**
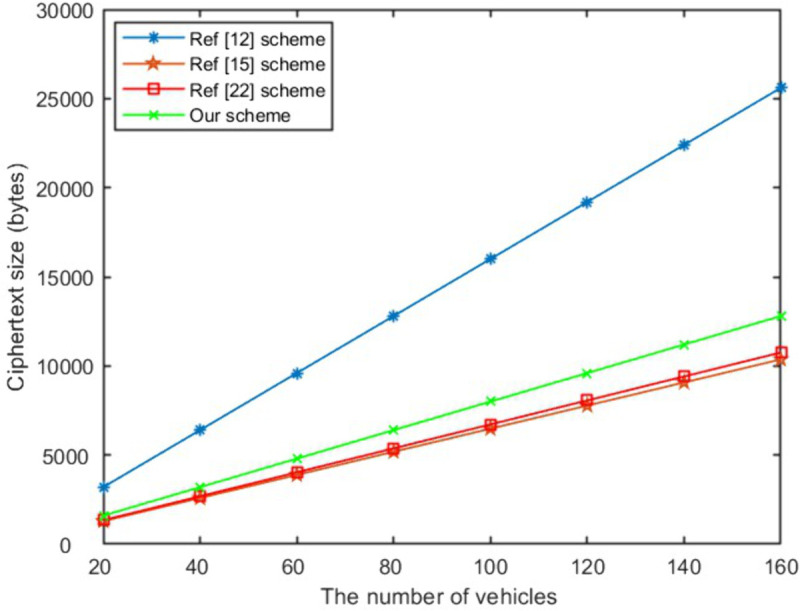
The size of ciphertext.

In the signcryption, unsigncryption and equality test stage, the computational efficiency of the proposed scheme in this paper is optimal compared with schemes [12,15,24]. When the number of receivers is 100, as presented in Fig 4, our scheme’s signcryption algorithm has increased computational efficiency by 58%, 35%, 70% respectively compared to exisited schemes. The unsigncryption computational time of the proposed work has increased by 71%, 10%, 67% respectively compared to above schemes as shown in Fig 5. It can be seen from Fig 6 that the efficiency of the proposed work has improved to 21% and 35% respectively compared to schemes [12,24] in equality test. As shown in Fig 7, Our scheme requires less ciphertext storage space than scheme [12], and higher rates than schemes [15] and [24]. Although the communication overhead of our scheme is not the lowest, it is a reasonable cost paid to improve computational efficiency.

## Conclusion

This paper proposes a secure data sharing scheme based on broadcast signcryption that supports multi-ciphertext equality test addressing security issues such as privacy data leakage. By combining broadcast signcryption and equality test techniques, the scheme ensures the confidentiality and unforgeability of privacy data. When data users want to retrieve ciphertext, they upload a trapdoor to the cloud server, which executes equality test algorithm to achieve efficient retrieval of ciphertext. The security of the scheme has been proven based on difficult problems in the random oracle model, and numerical experiment analysis have shown that the scheme has relatively low computational overhead and certain advantages in data center. Compared to some existing schemes, signcryption, unsigncryption, equality test algorithm of our scheme’s computational efficiency is superior, increasing by over 35%, 10% and 20% when the number of receivers is 100. While our scheme’s ciphertext storage overhead is not optimal, its computational efficiency is. In future work, we will focus on designing a broadcast signcryption scheme for heterogeneous communication environments and work to reduce storage overhead while maintaining high computational efficiency.
